# Mucosal and Systemic Immune Responses to Salmon Gill Poxvirus Infection in Atlantic Salmon Are Modulated Upon Hydrocortisone Injection

**DOI:** 10.3389/fimmu.2021.689302

**Published:** 2021-06-09

**Authors:** Marit M. Amundsen, Haitham Tartor, Kathrine Andersen, Karoline Sveinsson, Even Thoen, Mona C. Gjessing, Maria K. Dahle

**Affiliations:** ^1^ Department of Fish Health, Norwegian Veterinary Institute, Ås, Norway; ^2^ Patogen, Ålesund, Norway; ^3^ The Norwegian College of Fishery Science, UiT - The Arctic University of Norway, Tromsø, Norway

**Keywords:** salmon gill poxvirus, Atlantic salmon, antiviral immunity, cytotoxic cell, mucosal immunity, gill disease, virulence gene

## Abstract

Salmon Gill Poxvirus Disease (SGPVD) has emerged as a cause of acute mortality in Atlantic salmon (*Salmo salar L.*) presmolts in Norwegian aquaculture. The clinical phase of the disease is associated with apoptotic cell death in the gill epithelium causing acute respiratory distress, followed by proliferative changes in the regenerating gill in the period after the disease outbreak. In an experimental SGPV challenge trial published in 2020, acute disease was only seen in fish injected with hydrocortisone 24 h prior to infection. SGPV-mediated mortality in the hydrocortisone-injected group was associated with more extensive gill pathology and higher SGPV levels compared to the group infected with SGPV only. In this study based on the same trial, SGPV gene expression and the innate and adaptive antiviral immune response was monitored in gills and spleen in the presence and absence of hydrocortisone. Whereas most SGPV genes were induced from day 3 along with the interferon-regulated innate immune response in gills, the putative SGPV virulence genes of the B22R family were expressed already one day after SGPV exposure, indicating a potential role as early markers of SGPV infection. In gills of the hydrocortisone-injected fish infected with SGPV, *MX* expression was delayed until day 10, and then expression skyrocketed along with the viral peak, gill pathology and mortality occurring from day 14. A similar expression pattern was observed for Interferon gamma (*IFNγ*) and granzyme A (*GzmA*) in the gills, indicating a role of acute cytotoxic cell activity in SGPVD. Duplex *in situ* hybridization demonstrated effects of hydrocortisone on the number and localization of *GzmA*-containing cells, and colocalization with SGPV infected cells in the gill. SGPV was generally not detected in spleen, and gill infection did not induce any corresponding systemic immune activity in the absence of stress hormone injection. However, in fish injected with hydrocortisone, *IFNγ* and *GzmA* gene expression was induced in spleen in the days prior to acute mortality. These data indicate that suppressed mucosal immune response in the gills and the late triggered systemic immune response in the spleen following hormonal stress induction may be the key to the onset of clinical SGPVD.

## Introduction

The Salmon gill poxvirus (SGPV) can cause salmon gill poxvirus disease (SGPVD), often associated with acute, high mortalities in Atlantic salmon (*Salmo salar L.*) presmolts. This disease was first observed in 1995 at a Norwegian salmon hatchery (1), but was not shown to be associated with a virus until 2008, when viral particles were detected using transmission electron microscopy by Nylund et al. (2). In 2015 the full SGPV genome sequence was published by Gjessing et al. (1). SGPV has since then been detected in several salmon producing countries in Northern Europe, including Norway, Scotland and the Faroe Islands (3–5). SGPV have been associated with gill disease in farmed Atlantic salmon both at the freshwater presmolt stage and at the grow-out stage in the sea, although often in combination with multiple coinfecting agents in the sea (2). The most important clinical feature of SGPVD is its impact on the A. salmon respiratory system. Apoptotic gill epithelial cells detach during the acute phase of the infection, destroying large parts of the respiratory surface of the gills (1, 6). In the regenerating phase, epithelial cell hyperplasia leads to a thickened and less functional respiratory surface. Salmon infected with SGPV usually have no obvious pathology in other organs based on autopsy and histology (6), apart from accumulation of red blood cells in the spleen, a finding not yet clearly associated with SGPVD (6, 7).

Poxviruses represent a family of large enveloped DNA viruses with a double stranded linear genome, and can cause disease in several animal species, including mammals, birds, reptiles and fish (1). The virus family divides into two subfamilies; chordopoxvirus (ChPV) and entomopoxvirus (EnPV) which have vertebrates and insects as hosts, respectively (8). Phylogenetic analyses show that SGPV belongs to the family ChPV (1). SPGV is measured to a size of 360 x 270 x 250 nm (6) with a genome of more than 240 kb, putatively encoding 210 proteins (1). Many of the SGPV genes are homologous to other Chordopoxviruses, such as the genes that enable the virus to replicate and form viral envelopes. On the other hand, SGPV has several novel genes not found in other Chordopoxviruses, and as of today the function of these proteins are unknown (1).

One of the most severe human diseases, Smallpox, was caused by an orthopoxvirus named variola virus (VARV) (9). Smallpox disease is now eradicated through vaccination, and the initial “live” vaccine against VARV was the low pathogenic Vaccinia virus (VACV) (10), now a widely researched and often used model for other poxviruses. In 2011, Yang et al. published a genome-wide transcription map of early-, intermediate-, and late VACV genes (11), where many of the genes show high similarity to the SGPV genome (12). In SGPV, three genes (SGPV154, SPGV159 and SGPV162) are homologous to a putative virulence protein from the Variola virus, called *B22R* (1), and are here renamed to SGPV *B22R1*, *B22R2* and *B22R3*, respectively. The B22R genes are of particular interest when it comes to viral pathogenesis and interactions between the virus and the host immune system (1). Alzhanova et al. have shown that poxviruses with *B22R* encoding genes are associated with suppressed T-cell responses, and this ability to suppress T-cell activity might be directly related to virulence (13). SGPV *B22R1* has about the same length as the homologues in other poxviruses, while *B22R2* and *B22R3* are shorter, suggesting that these two genes have been truncated during the evolution of SGPV, and may have evolved by duplication events (1).

The mucosal immune system in the gills is characterized by several humoral and cellular immune mechanisms that interplay to protect the tissue from infection (14). Upon infection, both local populations of immune cells, including mucosal “innate” T-cells and IgT + B cells, as well as immune cells recruited through blood from specialized immune organs can help eradicate the infection (15). The host response to SGPV infection is only vaguely understood, and due to the many virus encoded genes, there are many putative host interaction mechanisms (1, 16). Like in mammals, viral infection in Atlantic salmon trigger the interferon system, leading to the secondary expression of a range of interferon response genes including myxovirus resistance gene (*MX1*) and interferon stimulated gene (*ISG15*), which are involved in inhibiting virus replication (17–19). Interferon regulated genes are previously shown to be strongly induced in gills from salmon in a natural SGPVD outbreak (7). Type 2 interferons like IFNγ are among others responsible for activating the cellular cytotoxic response in innate natural killer cells (NK cells) and adaptive CD8 + T cells (20), and are shown to be induced in SGPV-infected gills (7).

SGPV infection can turn out differently with regards to clinical manifestation and mortality. In the field, SGPV infection can vary from subclinical cases with low virus load to acute infection causing outbreaks with mortality up to 70% in severe cases (6). In the aquaculture industry, stress is one of the main factors that makes farmed fish more prone to disease (21). Chronic stress associated with high cortisol can be harmful to the fish, as it can have suppressive effects on immune responses to infection (22). Cortisol is a chronic stress hormone that can inhibit the immune system by preventing leukocyte migration to the local infection site and lead to a general reduction in the circulation of leukocytes and lymphocytes (23).

In a previously published experimental infection trial (24), we reported that fish exposed to SGPV developed mild gill pathology, but no mortality, whereas gross SGPVD with mortality was only seen when fish were injected with hydrocortisone prior to infection. Stress-induced suppression of the immune system was in that publication suggested as a potential reason for SGPVD development (24). Here, we explored the expression of SGPV genes and antiviral response genes in Atlantic salmon gills along the experimental infection course from the same trial (24), using RT-qPCR and RNAscope *in situ* hybridization. We identified putative SGPV virulence genes expressed early after infection, and compared immune responses in gills and spleen from infected fish groups with or without hydrocortisone injection.

## Materials and Methods

### Experimental SGPV Challenge Trial and Sampling

The samples used for analyses in this study originated from a previously published infection trial of SGPV in Atlantic salmon (24). Briefly, Atlantic salmon (n=220; average body weight 50 g) were divided into 4 groups and were allocated into 4 different tanks (55/each). Fish in two groups were exposed (E) to SGPV by cohabitating 55 naive fish (average weight 50g) with 10 fresh killed SGPV-infected fish (average weight 150g, average gill SGPV Ct level of 21,3) for 24 hours. The SGPV infected dead fish used for challenge originated from an ongoing hatchery outbreak in Northern Norway). Fish in the other two groups were left as uninfected negative controls (C). To study effects of cortisol stress on salmon susceptibility to SGPV, one exposed group and one control group had been injected intraperitoneally with hydrocortisone (Sigma-Aldrich, St. Louis, MO, USA) (H) in a depot matrix 24 hours prior to virus exposure. Fish in the other exposed and control groups had received a sham injection (S) of the depot matrix without hydrocortisone. The experiment lasted for 28 days, and gills and spleen samples (n=5) were collected from fish in all experimental groups at 1, 3, 7, 10, 14, 15, 21 and 28 days post exposure (dpe). The same tissue was divided and stored in RNAlater (Qiagen Inc., Valencia, CA, USA) for qPCR and RT-qPCR analysis, and in formalin for *in situ* hybridization. More detailed information on the trial is given in **Table 1** and Thoen et al., 2020 (24).

**Table 1:** A summary of trial information derived from (24). The trial lasted for 28 days after SGPV infection. Hydrocortisone injection was given 24 hours earlier (day -1). Dpe; Days post exposure.

	Control Sham group C.S	Control Hydrocortisone group C.H	Exposed (SGPV) Sham group E.S	Exposed (SGPV) Hydrocortisone group E.H
Gill SGPV (DNA) Ct level range in trial	ND	ND	Ct 22,4 – 29,6	Ct 17,8 – 30,9
Group mortality (%) at 28 dpe	0%	0%	0%	100%
Mean gill apoptosis score at 14 dpe (Severity range 0-3)	0	0	0,5	2,5
Plasma cortisol level at 1 dpe	3,7 ng/mL	43,8 ng/mL	3,8 ng/mL	120,2 ng/mL

### RNA Extraction and cDNA Synthesis

Total RNA was extracted from spleen and gills using RNeasy Mini kit (Qiagen) according to the manufacturer’s protocol, with minor modifications in the case of gills. Gill tissue (10-20 mg) was lysed in 500 μl of QIAzol (Qiagen) and homogenized using 5 mm steal beads with TissueLyser II (Qiagen) at 24.7 Hz for 2 x 5 minutes. After homogenization 100 μl of chloroform (VWR, Radnor, PA, USA) was added to each sample followed by centrifugation at 4°C and 11 300 rpm for 15 minutes. The upper aqueous phase was transferred to a new tube and mixed with one volume of 70% ethanol. The rest of the isolation procedure was performed according to the manufacturer’s protocol. After RNA extraction, RNase Out (Life technologies, Carlsbad, CA, USA) was added each sample. Finally, NanoDrop™ 2000 spectrophotometer (Thermo Scientific, Waltham, MA, USA.) was used to estimate purity and yield of RNA, and samples were stored at -80°C. Reverse transcription to synthesize cDNA was performed using 1 µg RNA input in a 20 μL reaction volume, using the QuantiTect Reverse Transcription kit (Qiagen) with gDNA elimination following the manufacturer’s instructions. After synthesis, the cDNA was diluted (1:20) to prepare working stock, using Nuclease-free free water. The diluted and the original samples were stored at -20°C until further use.

### Gene Expression Analysis

We used RT-qPCR to analyze the expression of SGPV genes in gills (*B22R1, B22R2, B22R3, D12L, A1L, A2L, A7L, A28L, F9L, G1L*), and the Atlantic salmon genes *MX1*, *ISG15* (gills), *CD8α*, *CD4*, *IFNγ* and *GzmA* (in gills and spleen) in fish collected from the four experimental groups (C.S, C.H, E.S, and E.H), at 1, 3, 7, 10, 14, 15, 21 and 28 dpe. The A. salmon elongation factor 1α (*EF1α*) gene was used as a housekeeping gene. Primer information is given in **Supplementary Table 1**. The amplicon length for each RT-qPCR product was controlled using 2100 Bioanalyzer with DNA 1000 kit (Agilent Technologies, Santa Clara, CA.USA), shown in **Supplementary Image 1**.

The RT-qPCR was performed using CFX384 Touch Real-Time PCR Detection System (Bio-Rad Laboratories, Germany). Each sample was analyzed in duplicate, using a total reaction volume of 10 µL per well (5 ng cDNA, primers at 10 µM, 2 µL nuclease-free water and 5 µL of 2 x SsoAdvanced™ Universal SYBR^®^ Green Supermix (Bio-Rad Laboratories)master mix). No-template control (H_2_O) and no reverse transcriptase control (NRT) were included on each plate as negative controls.

The following thermocycling conditions were used: initial denaturation (30 s at 95°C) followed by 40 cycles of denaturation (15 s at 95°C) and annealing/extension (30 s at 60°C). A melting curve was made by measuring the fluorescence during a temperature range of (55-95°C) to confirm the specificity of the final amplicon in each reaction. Quantification cycles (Cq) for every reaction was measured and RT-qPCR data were analyzed using the CFX Manager software version 3.1.1621.0826 (Bio-Rad Laboratories). All gene expression values were then normalized to *Ef1α* values, resulting in -ΔCt values (Ct target genes – Ct *EF1α*). Raw data for all RT-qPCR runs are given in **Supplementary Table 2** (SGPV genes), **Supplementary Table 3** (Salmon genes gill) and **Supplementary Table 4** (Salmon genes spleen). Variation in *Ef1α* levels in gill and spleen samples is shown in **Supplementary Image 2**. Samples with *EF1a* Ct values > 1,5 Ct difference from sample set mean) were removed from the data set.

### DNA Extraction and qPCR for SGVP

DNA was extracted from gills using QIAcube and QIAamp DNA mini kit (Qiagen) as described Thoen et al., 2020 (24). DNA from gills was analyzed using qPCR (probe assay) from both E.S and E.H group at 1, 3, 7, 10, 14, 15, 21 and 28 dpe, and the same assay was used to analyze cDNA from spleen from both E.S and E.H group at 14 dpe. Each sample was analyzed in duplicate, using a total reaction volume of 10 µl per well (50 ng DNA, primers and probe at 10 µM, MgCl_2_ at 50 mM, 1,6 µl nuclease-free water and 5 µl UDG platinum supermix (Thermo Scientific)). The following thermocycling conditions were used: UDG incubation (2 min at 50°C), UDG inactivation (15 min at 95°C) followed by 94 °C/15 s, 55 °C/30 s and 72 °C/15 s.

### In *Situ* Hybridization

In the current study, both the single-plex and duplex variants of RNAscope protocol was used. RNAscope^®^ 2.5 HD Singleplex Red Chromogenic Reagent Kit (Advanced Cell Diagnostics Inc. Newark, CA, USA) was used for the detection of SGPV-B22R1 and D13L transcripts in Atlantic salmon gills at early time points after virus exposure. For this purpose, serial sections from fish gills in the E.S group at 1 dpe (n=3), positive control at 3 dpe, plus uninfected negative control were prepared for probe hybridization as previously described by Thoen *et al. (* 24). Briefly, the slides were deparaffinized, rehydrated, and endogenous peroxidase was blocked using hydrogen peroxide. The sections were then boiled in target retrieval buffer for 15 min and incubated with protease at 40°C for 15 min. The sections were hybridized with probes (**Supplementary Table 1**) targeting B22R1 and D13L genes of SGPV with the same amount of probe used for each section. Fast Red chromogenic substrate was used to visualize the signal.

RNAscope^®^ 2.5 HD Duplex Detection Chromogenic kit (Advanced Cell Diagnostics) was used for simultaneous detection of SGPV-D13L and salmon GzmA in Atlantic salmon gills from all groups at 7 dpe (n_(E.S)_= 3, n_(E.H)_= 3, n_(C.S)_= 1, n_(C.H)_= 1) along with one section from the E.S group and the E.H group at 14 dpe. Spleen sections included were from 14 dpe (n_(E.S)_= 3, n_(E.H)_= 3, n_(C.S)_= 1, n_(C.H)_= 1). Slides were prepared for probe hybridization as in the single-plex assay. After that, probes targeting SGPV-D13L, and Atlantic salmon GzmA (**Supplementary Table 1**) were combined and hybridized to the prepared sections. Amplification (Amp1 - Amp10), and washing steps were completed according to the manufacturers’ protocol. Signals were developed using red substrate for *GzmA* and green substrate for SGPV (*D13L*). All slides were counterstained for 30 seconds using Mayer’s hematoxylin (Chemi Teknik, Oslo, Norway) diluted 1:1 in distilled water, and mounted with VectaMount (Vector Labs, Burlingame, CA). An overview of all sections used for both single-plex and duplex-assays are listed in **Supplementary Table 5**.

### Statistics

The RT-qPCR data were analyzed in Graphpad Prism 8.0.2. Ct values from A salmon gene expression were normalized to Ct levels of *EF1α*, and relative gene expression was calculated using the 2^-ΔΔCt^ method. Statistically significant differences between groups at the time points of focus for our analyses, were calculated using a two-tailed non-parametric Mann-Whitney test. P-values are given in the figures in question, and between additional groups in **Supplementary Table 6**.

Spearman’s rank correlation (Spearman r) was used to calculate correlation between expression of *MX1* and amount of virus (DNA) in gill tissue.

## Results

This study is based on a previously published experimental SGPV infection trial (24). **Table 1** summarizes the background data for the study groups. We here focus on the factors underlying the mortality difference associated with SGPV- infected groups with or without hydrocortisone injection (E.S and E.H groups)

### Gene Expression of SGPV B22R Represents an Early Marker for SGPV Infection

**Figure 1:**
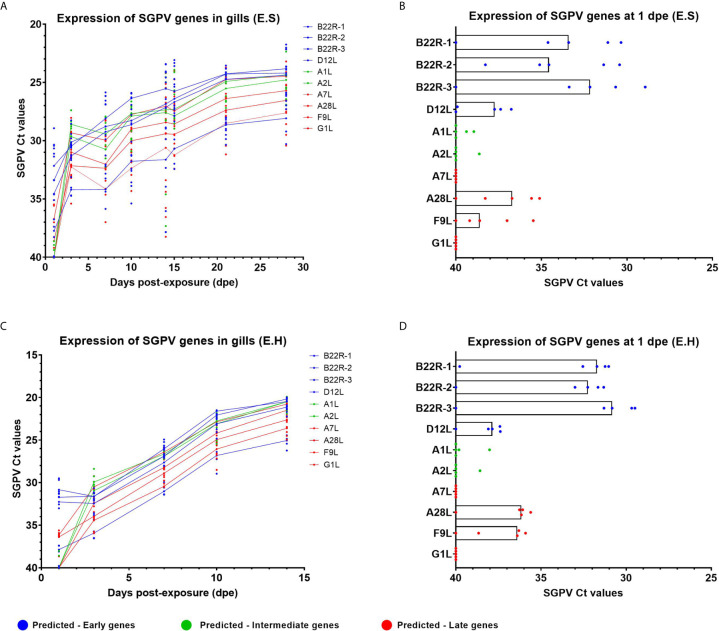
Expression of SGPV genes predicted as early-, intermediate- and late based on the Vaccinia virus (11). Each dot represents data from one individual salmon and the solid line represents the median value for each group. Predicted early genes are marked in blue, predicted intermediate genes in green and predicted late genes in red. **(A)** Expression of SGPV genes for the E.S group from day 1 to day 28 in the experimental trial. **(B)** Highlight from day 1 post-exposure for the E.S group. **(C)** Expression of SGPV genes for the E.H group from day 1 to day 14 in the experimental trial. **(D)** Highlight from day 1 post-exposure for the E.H group. E.S, SGPV-exposed sham injected group; E.H, SGPV-exposed hydrocortisone-injected group.

In the previous report on this infection trial (24), replication of SGPV was analyzed in gills using a qPCR assay targeting the D13L gene sequence in the SGPV DNA genome (1). The gene expression pattern of individual SGPV genes had not been studied previously, and RT-qPCR was performed to investigate the expression of SGPV genes that were predicted to belong to early poxviral genes (*B22R1, B22R2, B22R3, D12L)*, intermediate genes *(A1L, A2L)*, and late genes (*A7L, A28L, F9L, G1L)*, based on previous reports from research on the Vaccinia poxvirus (11). Gills from salmon in both infected groups, E.S, and E.H, were investigated (**Figure 1**, **Supplementary Table 2**). The expression of the selected SGPV genes ​​followed a similar trend throughout the experimental trial in both the E.S and E.H groups, although with higher expression in the E.H. group (e.g. at 14 dpe for the B22R1 gene: Median in the E.H group: Ct 20,2 +/- 1,4, Median in the E.S group: Ct 26,9 +/- 14,4) (**Figures 1A-C**). These group differences are in line with differences in SGPV levels based on qPCR targeting the genome (24). All three *B22R* genes, predicted for early expression, showed higher expression/lower Ct values at day 1 compared to all other SGPV genes (**Figures 1B-D**), with somewhat higher median expression levels in the E.H. group (Median: B22R1; Ct 31,7 [+/- 8,8], B22R2; Ct 32,1 [+/- 8,7], B22R3; Ct 30,8 [+/- 10,5]), compared to the E.S. group (Median: B22R1; Ct 33,4 [+/- 9,7], B22R2; 33,9 [+/- 7,8, B22R3; Ct - 32,2 [+/- 11,1]).

**Figure 2:**
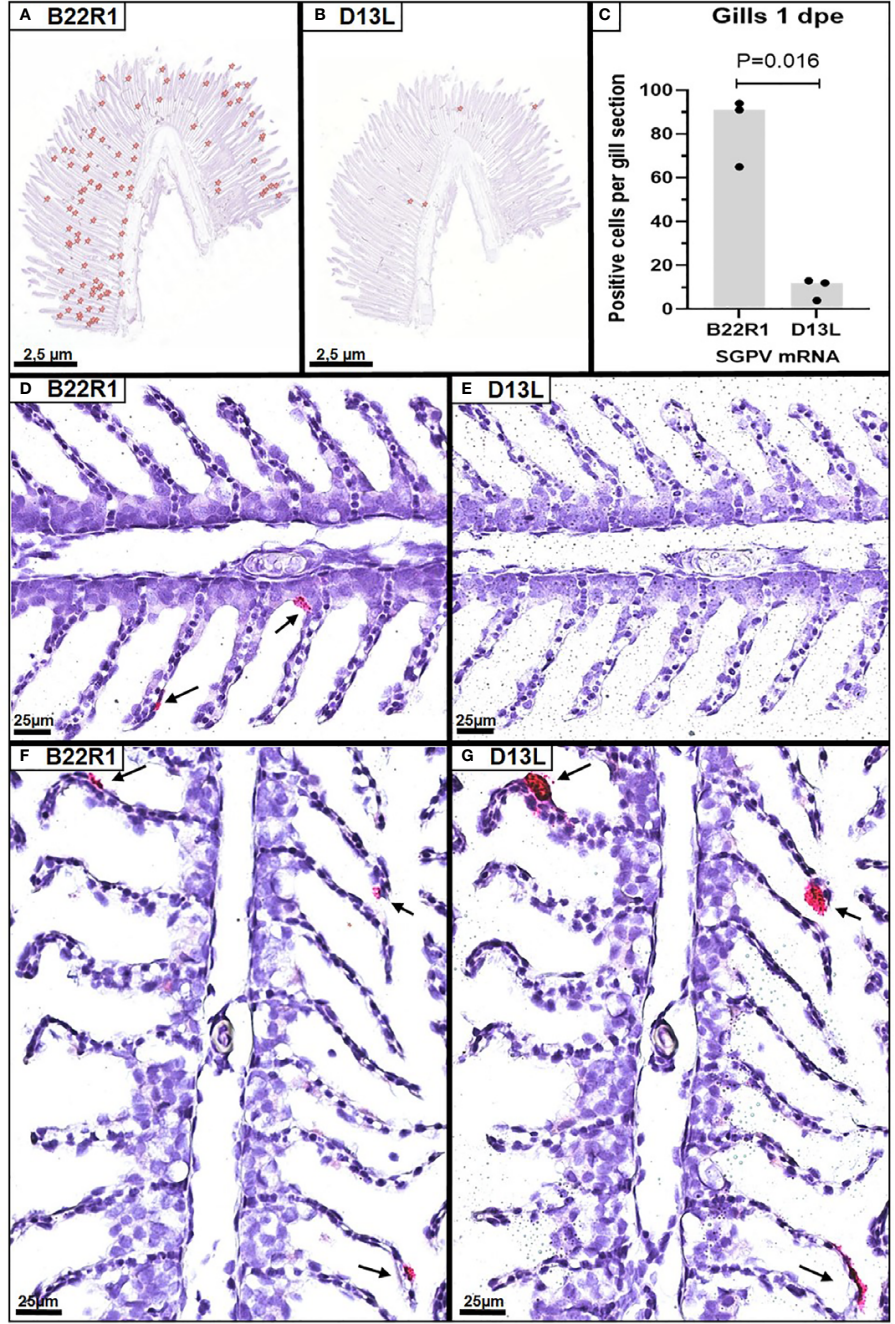
Singleplex *in situ* hybridization (RNAscope) showing detection of SGPV *B22R1* and *D13L* transcripts in gills from Atlantic salmon at 1 and 3 days after exposure to SGPV (dpe). Whole gill serial sections from 1 dpe (n=3) were scanned for staining of B22R1 and D13L RNA, as marked **(A, B)**, revealing significant differences in detection of SGPV-positive cells **(C)**. Images of the SGPV-exposed sham injected (E.S) group from 1 dpe stained with *B22R1* probe **(D)** and D13L probe **(E)**. Serial section from the E.S group from 3 dpe stained with *B22R1* probe **(F)** and *D13L* probe **(G)**.

*In situ* hybridization using probes targeting SGPV *B22R1* and *D13L* was performed on selected paraffin-embedded gills from the E.S group at 1dpe (N=3 in each group). As controls, two selected paraffin-embedded gills from E.S. group at 3 dpe were also analyzed. Parallel sections from each gill were stained with the B22R1 probe and the D13L probe, respectively. Images of all sections analyzed are included in **Supplementary Image 3**. Positive staining were counted in the whole gill sections from 1 dpe (**Figures 2A, B**), and quantified for comparison between B22R1 and D13L staining (**Figure 2C**). At 1 day post exposure, epithelial cells appeared with normal morphology, and significantly more epithelial cells stained for B22R1 RNA compared to D13L RNA at this time point (**Figures 2D, E** and **Supplementary Image 3**). At 3 dpe, however, staining for both *B22R1* and *D13L* transcripts were revealed in the same location (**Figures 2F, G** and **Supplementary Image 3**).

### Antiviral Immune Genes Are Upregulated in Gills Infected With SGPV

**Figure 3:**
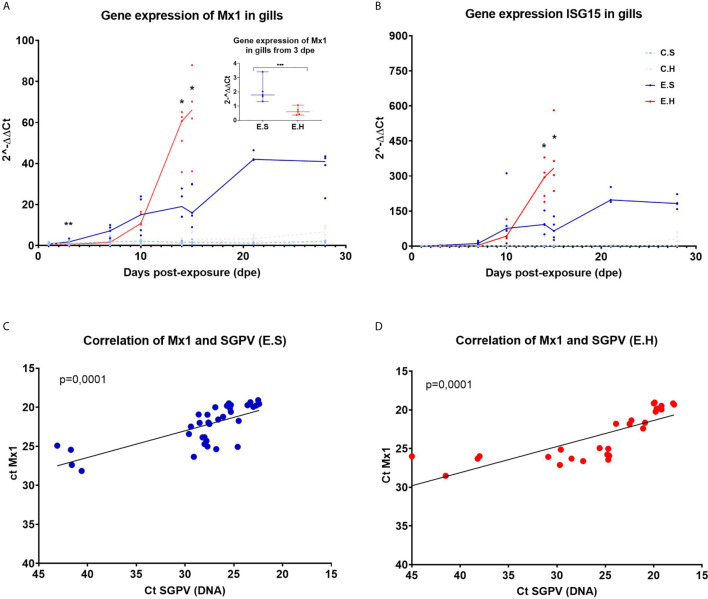
Gene expression of *Mx1* and *ISG15* in gills from Atlantic salmon from the experimental trial. Each dot represents data from one individual salmon and the solid line represents the median value for each group. The significant differences are calculated between the two infected groups. **(A)** Gene expression of *MX1* for all groups from day 1 to day 28 in the experimental trial. (**A**-insert) Significant differences between gene expression of *MX1* in the two infected groups at 3 dpe. **(B)** Gene expression of *ISG15* for all groups from day 1 to day 28 in the experimental trial. **(C)** Ct correlation of *MX1* and SGPV DNA (D13L) in the E.S group. **(D)** Ct correlation of *MX1* and SGPV DNA (D13L) in the E.H group. *P < 0.05, **P < 0.01, *** P < 0.001. C.S, Uninfected control sham injected group; C.H, Uninfected control hydrocortisone-injected group; E.S, SGPV-exposed sham injected group; E.H, SGPV-exposed hydrocortisone-injected group.

Expression of the IFN regulated innate antiviral genes *MX1* and *ISG15* were analyzed by RT-qPCR in gills from fish in all experimental groups (**Figure 3**, **Supplementary Tables 3** and **6**). The innate antiviral immune response was monitored in gills only since SGPV were not detected in blood, and only detected at low levels in spleen and head kidney in some individuals (24).

*MX1* expression was induced from 3 dpe in response to SGPV infection and stayed elevated throughout the experimental period (**Figure 3A**). The *MX1* transcript level in the E.H group was significantly lower at day 3 (p = 0.0079) (**Figure 3A** insert) compared to the E.S. group, and was not induced until day 10 post exposure, and then strongly upregulated along with the viral peak. For both E.H and E.S groups, there is a statistically significant correlation between the expression of *MX1* and load of SGPV based on SGPV genome detection with qPCR (**Figures 3C, D**).

Gene expression of *ISG15* follow the same trend as *MX1* with a gradual upregulation of the gene for the E.H and E.S groups, up to the viral peak at 14 dpe (**Figure 3B**). However, there were no significant differences in *ISG15* levels between the exposed groups prior to day 14. The gene expression in the two control groups remained stably low throughout the course for both *MX1* and *ISG15*.

### Effect of Hydrocortisone on CD4 and CD8 Gene Expression in Gill and Spleen

**Figure 4:**
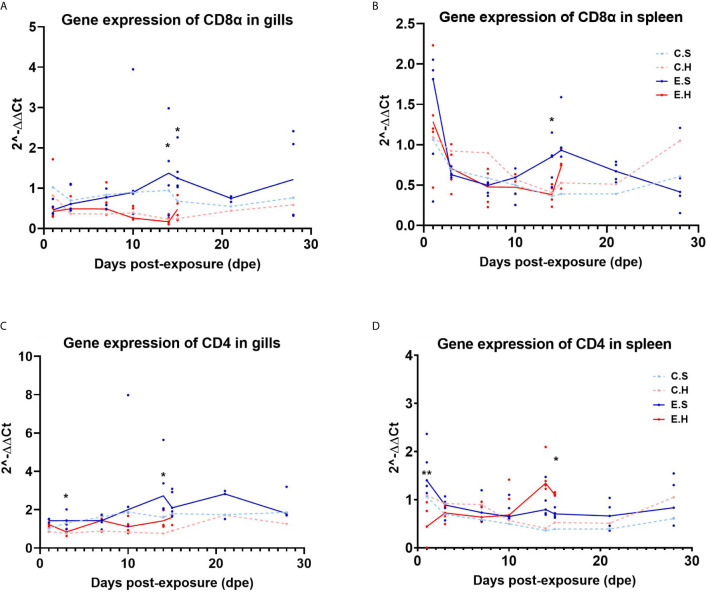
Gene expression of CD8α in gills **(A)** and spleen **(B)** and gene expression of *CD4* in gills **(C)** and spleen **(D)** from the entire SGPV infection trial. Both median (line) and individual data (dots) are shown from the SGPV-exposed groups. Control groups are only shown with median value. Significant differences are calculated between the two infected groups, E.S and E.H. *P< 0.05, **P< 0.01. C.S, Uninfected control sham injected group; C.H, Uninfected control hydrocortisone-injected group; E.S, SGPV-exposed sham injected group; E.H, SGPV-exposed hydrocortisone-injected group.

The T cell marker transcripts *CD4* and *CD8α* were analyzed in gills and spleen from the entire challenge experiment to assess the local and systemic regulation of T cells in the two different organs during the course of SGPV infection (**Figure 4**, **Supplementary Tables 3, 4** and **6**). There was a significantly lower *CD8α* transcript level in the E.H group in both gills and spleen at 14 dpe (**Figures 4A, B**). The gene expression of CD4 in gills also appeared suppressed in the hydrocortisone injected group (**Figure 4C**). In the spleen, *CD4* gene expression was significantly lower in the E.H group than the E.S group at 1 dpe, and higher in the E.H group when this group suffered from disease mortality (24).

### Expression of Cytotoxic Immune Effector Genes

**Figure 5:**
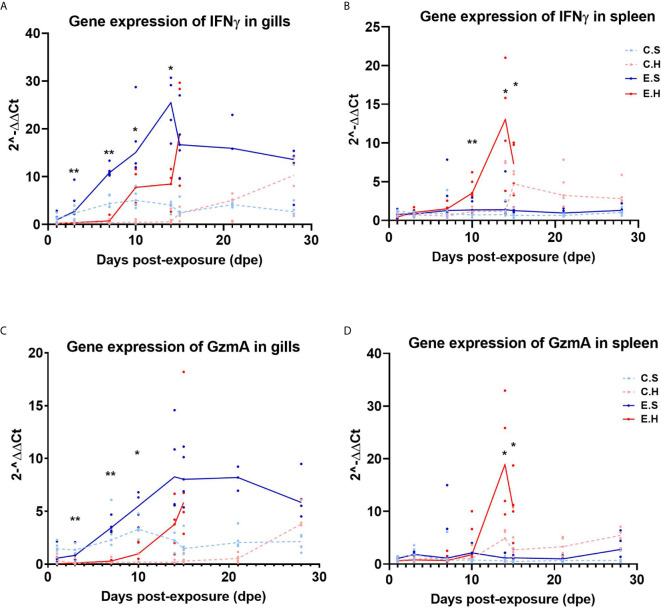
Gene expression of IFNγ in gills **(A)** and spleen **(B)** and GzmA in gills **(C)** and spleen **(D)** from the experimental trial. Each dot represents data from one individual salmon and the solid line represents the median value for each group. The significant differences for all figures are measured between the two infected groups. *P<0.05, **P< 0.01. C.S, Uninfected control sham injected group; C.H, Uninfected control hydrocortisone-injected group; E.S, SGPV-exposed sham injected group; E.H, SGPV-exposed hydrocortisone-injected group.

To monitor cytotoxic immune activity, *IFNγ* and *GzmA* were analyzed in gills and spleen from all fish in the challenge experiment (**Figure 5**, **Supplementary Table 6**). In the gills, there was a significantly higher expression of *IFNγ* from 3 dpe onwards in the E.S group, peaking at 14 dpe (**Figure 5A**, **Supplementary Table 2**). In contrast, *IFNγ* gene expression was not induced in spleen in the E.S group, but strongly in the E.H group from day 10 onwards (**Figures 5B**, **Supplementary Table 3**). Gene expression of *GzmA* shows the same trend as *IFNγ* for all groups in both gill (**Figure 5C**) and spleen (**Figure 5D**).

**Figure 6:**
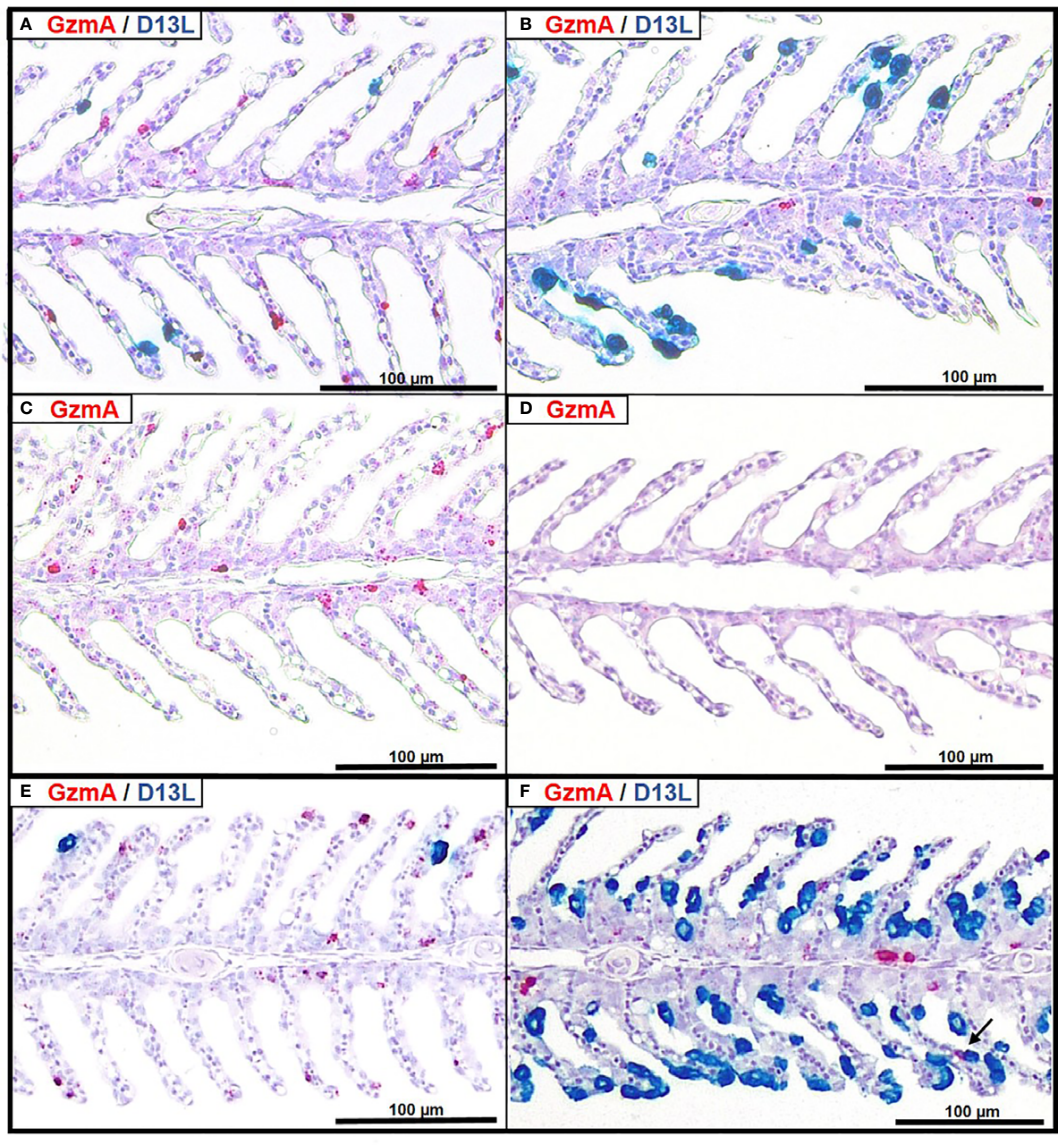
Duplex *in situ* hybridization (RNAscope) demonstrating the distribution of SGPV (D13L, blue staining) and salmon GzmA (red staining) in gills from Atlantic salmon. **(A)** E.S group at 7 dpe. **(B)** E.H group at 7 dpe. **(C)** C.S group at 7 dpe. **(D)** C.H group at 7 dpe. **(E)** E.S group from 14 dpe. **(F)** E.H group at 14 dpe. Arrow show interaction between GzmA expressing cell and SGPV-infected cell.

To explore the relation between *GzmA* expression and SGPV infected cells in the gills, duplex *in situ* hybridization was performed on selected paraffin-embedded gill sections from all groups at 7 dpe, in addition to 1 section from the E.S and E.H group at 14 dpe. *GzmA* positive cells dominated over SGPV infected cells in the E.S group for both 7 and 14 dpe (**Figures 6A, E**, **Supplementary Image 4**). In the E.H group, however, the number of gill epithelial cells staining positive for SGPV were dominant compared to the few cells staining positive for *GzmA* (**Figures 6B, F**). In the control groups, no staining for SGPV were observed, but moderate staining for *GzmA* was seen in the C.S group (**Figure 6C**). In the C.H group, only a few *GzmA* positive cells were observed in the entire gill section (**Figure 6D**). In some areas, it was possible to visualize *GzmA* positive cells next to with SGPV infected cells (**Figure 6F**).

**Figure 7:**
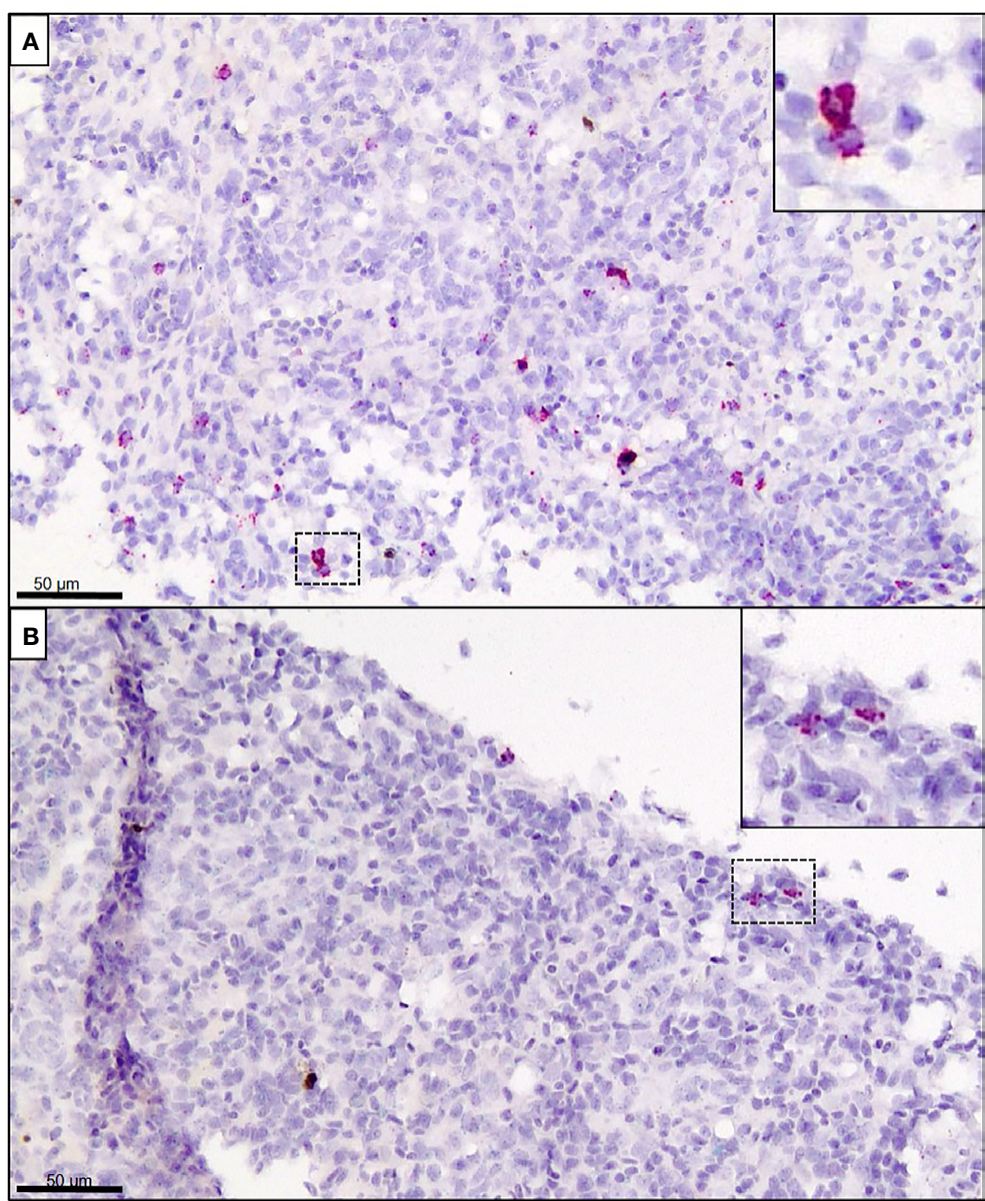
*In situ* hybridization (RNAscope) targeting GzmA (stained in red) in spleen from Atlantic salmon at 14 dpe. **(A)** SGPV-exposed hydrocortisone-injected (E.H) group. **(B)** SGPV-exposed sham-injected (E.S) group.

In the spleen at 14 dpe, only one individual showed trace staining of SGPV, whereas a moderate number of cells were stained for *GzmA* in the E.H group (**Figure 7A**, **Supplementary Image 5**). In comparison, only a few cells showed positive staining for *GzmA* in the E.S group (**Figure 7B**). In the C.H group, some cells with positive staining for GzmA were found, whereas no staining for GzmA were observed in the C.S group (**Supplementary Image 5**).

## Discussion

We aimed in this study to explore SGPV gene expression and the host immune response to infection, to understand the mechanisms behind the previously reported hydrocortisone-mediated triggering of SGPV replication, SGPVD onset and mortality observed in experimental trials [(24), **Table 1**].

Large DNA viruses like SGPV contain a wide range of genes involved in host interaction and can be in a complex interplay with the host immune system (14, 25). We still know next to nothing about how SGPV interacts with the mucosal immune system in Atlantic salmon gills, and have to extrapolate from research on other poxviruses.

Poxvirus replication is cytoplasmatic, and the transcriptional machinery is encoded by the virus. In vaccinia, the replication is determined as a three phase cycle of early, intermediate and late gene expression, controlled by stage specific transcription factors (26). The early genes are regulated by transcription factors carried by the infecting virus and expressed prior to genome replication, encoding proteins essential for replication and host interaction (26). Intermediate and late genes are generally expressed during and post-replication, respectively, and encode most of the structural proteins to form new viral particles. When exploring gene expression data from a range of SGPV genes predicted as early, intermediate and late based on previous studies on the Vaccinia virus (11), we found no obvious differences in expression throughout the experiment for the infected groups, with the exception of the B22R gene family. The lack of variation is most likely due to the limited set of pre-replication samples available from the study. We had previously based on qPCR data determined the onset of SGPV genome replication to day 3 in this infection trial (24), and had only one sampling point prior to this (day 1).

However, we found that the predicted early genes encoding three paralogues of *B22R* (1–3) were higher expressed at day 1 compared to other SGPV genes, and this points to B22R transcripts as markers of early SGPV subclinical infection. SGPV replication is shown earlier to be boosted by hydrocortisone (24), and we also found a trend towards *B22R1* being higher expressed in the E.H group than in the E.S. group, and a steeper increase in expression of all SGPV genes towards the onset of mortality 14 dpe in the E.H group. The *in situ* hybridization of *B22R1* probes compared to D13L probes also demonstrated that B22R was expressed in significantly more gill epithelial cells from day 1, whereas D13L expression were much lower expressed until day 3. Surprisingly, SGPV genes predicted to be expressed as late genes (A28L and F9L), appeared to be expressed at low levels day one, in contrast to genes predicted as intermediate (A1L and A2L). This could indicate that the SGPV replication cycle is somewhat divergent from VACV replication.

Although we identified the B22R genes as early markers of subclinical SGPV infection, the limited set of available samples from this study were not suited to identify other SGPV genes as early, intermediate or late. Further work to explore the SGPV replication cycle is needed, and more excessive early sampling should be considered in future trials. A cell line susceptible for SGPV infection would make an excellent tool for future study of the SGPV infection cycle and the function of SGPV encoded host interacting proteins. Much is unknown about the SGPV replication cycle, and effects of stress along with other external factors like temperature, salinity and the gill mucus microbiome would be valuable to explore. The *in situ* hybridization of *B22R1* compared to *D13L* transcripts also demonstrates that *B22R1* is expressed in epithelial cells with normal morphology at day 1 after exposure, whereas *D13L* expression is predominantly detected in apoptotic, detaching cells from day 3.

The B22R gene encodes a large transmembrane protein, and its role have been associated with virulence in other poxviruses (9). Knocking out the B22R homologue in the monkey poxvirus (MXPV) led to lower viremia and less mortality, and the viruses lacking the B22R homologue were associated with higher T-cell activation after infection, suggesting a role of B22R in suppression of T cell activity (9). *B22R* was in that study reported to interfere after the T-cell receptor (TCR) has bound the antigen presenting MHC, and suggested to inhibit the signaling pathway downstream of TCR binding. Since B22R is a transmembrane protein, the T-cells are thought to be inhibited through cell-cell contact (13). Given this putative immune suppressive role of B22R paralogues, or at least the full length B22R1 in SGPV, the expression of this gene may prove to predict the putative outcome of infection at an early stage.

In an earlier study of the gill transcriptome of salmon presmolts infected with SGPV during a natural outbreak of SGPVD (7), we could not observe any obvious recruitment of T-cells to gills after infection, and observed an early suppression of transcript markers of innate T-cell recruitment and activity, including interleukin (*IL)-22*, the chemokine *CCL20* and T-cell receptor *(TCR) Fcγ* (7, 13). This effect may be due to chronic stress or to SGPV virulence factors like B22R acting on T cell recruitment and/or activation (13). In contrast, strong innate interferon-regulated gene expression was observed in salmon gills after SGPV infection, including upregulation of *Mx1* and *ISG15* (7). This study showed a correlated increase in *MX1* expression and SGPV replication for both infected groups, of which the E.H group showed a steeper increase from 10 dpe, few days prior to SGPVD mortality. Previous studies have shown that high cortisol levels can inhibit the innate immune response, causing a delay in *MX1* expression (27), also shown using cortisol implants in salmonids (27). At 3dpe, *MX1* expression was significantly higher in the E.S group, supporting previous studies showing cortisol-mediated suppression of *MX1* expression. Whether the reduced *MX1* expression early in the E.H. group determines the more dramatic infection and disease course in this group is unknown. It is also unclear if this is a direct effect of cortisol, or regulated by SGPV host interacting factors with higher expression in this group. Interferon inhibitory factors have been reported for other poxviruses (28), and also predicted for SGPV (7). It should be noted that direct inhibitory effects on poxvirus replication have been reported for ISG15 (29), but so far not for Mx.

Previous studies in fish have shown that cortisol can inhibit the immune system by down-regulating the number of circulating leukocytes and lymphocytes, as well as by preventing leukocytes from migrating to the area of ​​inflammation (23, 30). The results of this study indicated a similar effect based on gene expression of *GzmA* and *IFNγ* in gills and spleen. In the SGPV infected E.S group, results indicated a recruitment of cytotoxic cells to gills, based on a significant increase in the cytotoxic T-cell activity markers *IFNγ* and *GzmA.* In contrast, hydrocortisone-injected fish from the E.H group appear to lack the mobilization of cytotoxic cells to the gills in the early disease phase, and just immediately prior to SGPVD mortality, immune activity was induced in both gills and spleen, most likely due to a systemic immune response in the fish. The lower immune activity at the local site of infection was also seen in the intestine of A. salmon treated with hydrocortisone prior to infection with IPNV (30).

Notably, a significant difference in cytotoxic gene expression in gills was seen already 3 days post exposure. Since this is early for an adaptive T-cell response, it is a possibility that *IFNγ* and *GzmA* production are associated with innate cytotoxic cells, like NK cells or NKT-cells. NK cells form part of the first-line defense against virus-infected cells, and have previously been reported to be inhibited under chronic stress (31). Furthermore, IFNγ has been shown to enhance respiratory activity and nitric oxide production (32, 33), which suggests that inhibited IFNγ production in the E.H. group may be one of the contributing factors to the onset of clinical disease.

In spleen, *GzmA* and *IFNγ* were strongly upregulated after 10 days in the E.H group. No clear upregulation of *CD8α* was detected, but the spleen contains many T cells in the normal state and it cannot be ruled out that the CD8 T cells are involved in the production of *GzmA* and *IFNγ*. The upregulation of *GzmA* and *IFNγ* in the spleen few days prior to acute SGPV indicate that the disease is associated with a systemic immune response in contrast to the controlled local response in the E.S. group. A direct regulation of granzyme A expression by cortisol have been reported, with a putative role in immune cell apoptosis in response to stress (34). However, this would be expected seen in the early phase after hydrocortisone injection, and is not a likely mechanism here. This systemic response does not appear to be triggered by a systemic infection by SGPV, as no virus was detected in blood or spleen, as seen in the duplex ISH in fig 7 and reported in Thoen et al., 2020 (24). From previous field outbreaks, signs of hemophagocytosis in the spleen have been observed during histopathological examinations (1, 12), which could be a sign of a systemic response. Taken together, this study has revealed that gene expression of the putative virulence gene B22R is a potential early marker of SGPV infection, detectable already after one day, and that hydrocortisone injection suppresses antiviral immune responses to SGPV during early infection. The immune suppression involves local innate and cytotoxic T-cell associated antiviral immune activity in gills, leading to induced SGPV replication. Instead, a boost in innate and cytotoxic immune response occurs both locally in gills and systemically in the uninfected spleen a few days prior to the onset of acute SGPVD and mortality. This indicates that mortality may be caused by a systemic immune response.

## Data Availability Statement

The datasets presented in this study can be found in the **Supplementary Material**.

## Ethics Statement

The animal study was reviewed and approved by the Norwegian Animal Research Authority (FOTS IDs: 15042).

## Author Contributions

MD and MG planned the study. ET and MG designed the challenge trial. MA, HT, KS, ET, MG, and MD sampled from the trial. MA prepared RNA, cDNA, performed RT-qPCR analyses, and analysed data under supervision by MD. KA characterized B22R genes and prepared analysis tools. MA performed the *in situ* hybridization under supervision by HT and MG. MA prepared the figures and the first draft of the manuscript. HT, MG, and MD assisted in data interpretation and manuscript writing. All authors contributed to the article and approved the submitted version.

## Funding

This work was funded by the Norwegian Research Council, grant numbers 267491 (SALPOX) and 303415 (IMMUNOPOX). Open access publishing was funded by the Norwegian Veterinary Institute.

## Conflict of Interest

ET was employed by Patogen.

The remaining authors declare that the research was conducted in the absence of any commercial or financial relationships that could be construed as a potential conflict of interest.
