# Supplementary figures and images for: Mucosal and Systemic Immune Responses to Salmon Gill Poxvirus Infection in Atlantic Salmon Are Modulated Upon Hydrocortisone Injection
Source: Front Immunol. 2021 Jun 9;12:689302. doi: 10.3389/fimmu.2021.689302 (PMC8221106; doi:10.3389/fimmu.2021.689302)

Bioanalyzer - Gel Report for SGPV qPCR assays

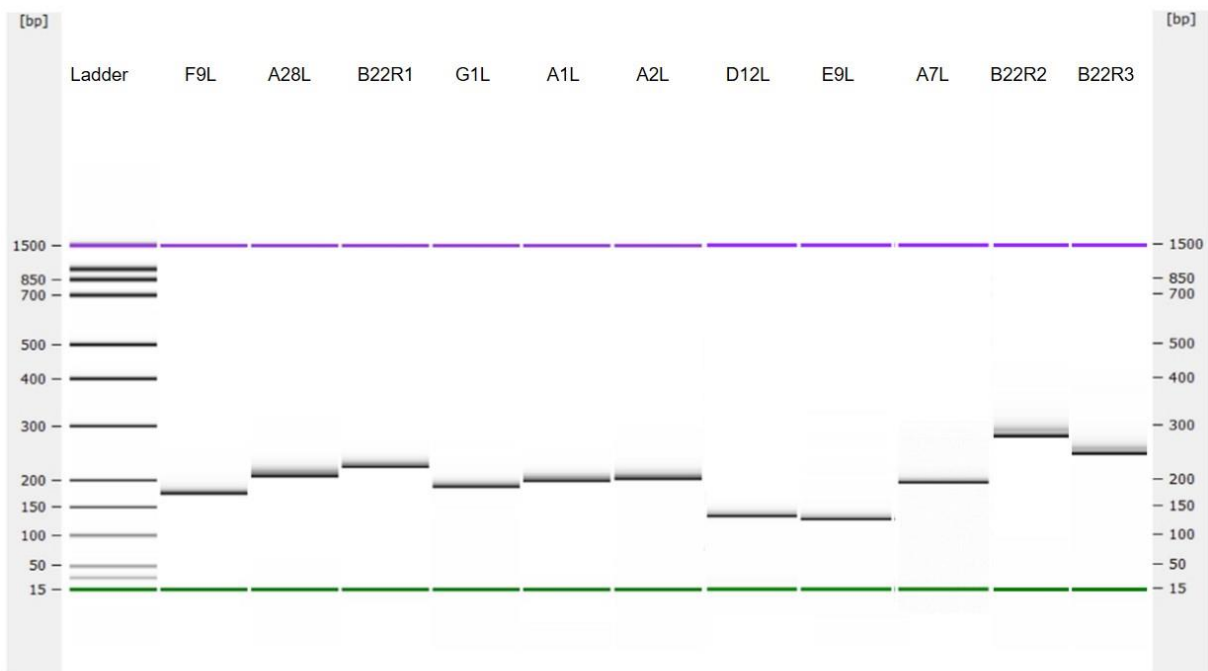

Supplement: Supplementary file 1 [file Image_1.pdf]

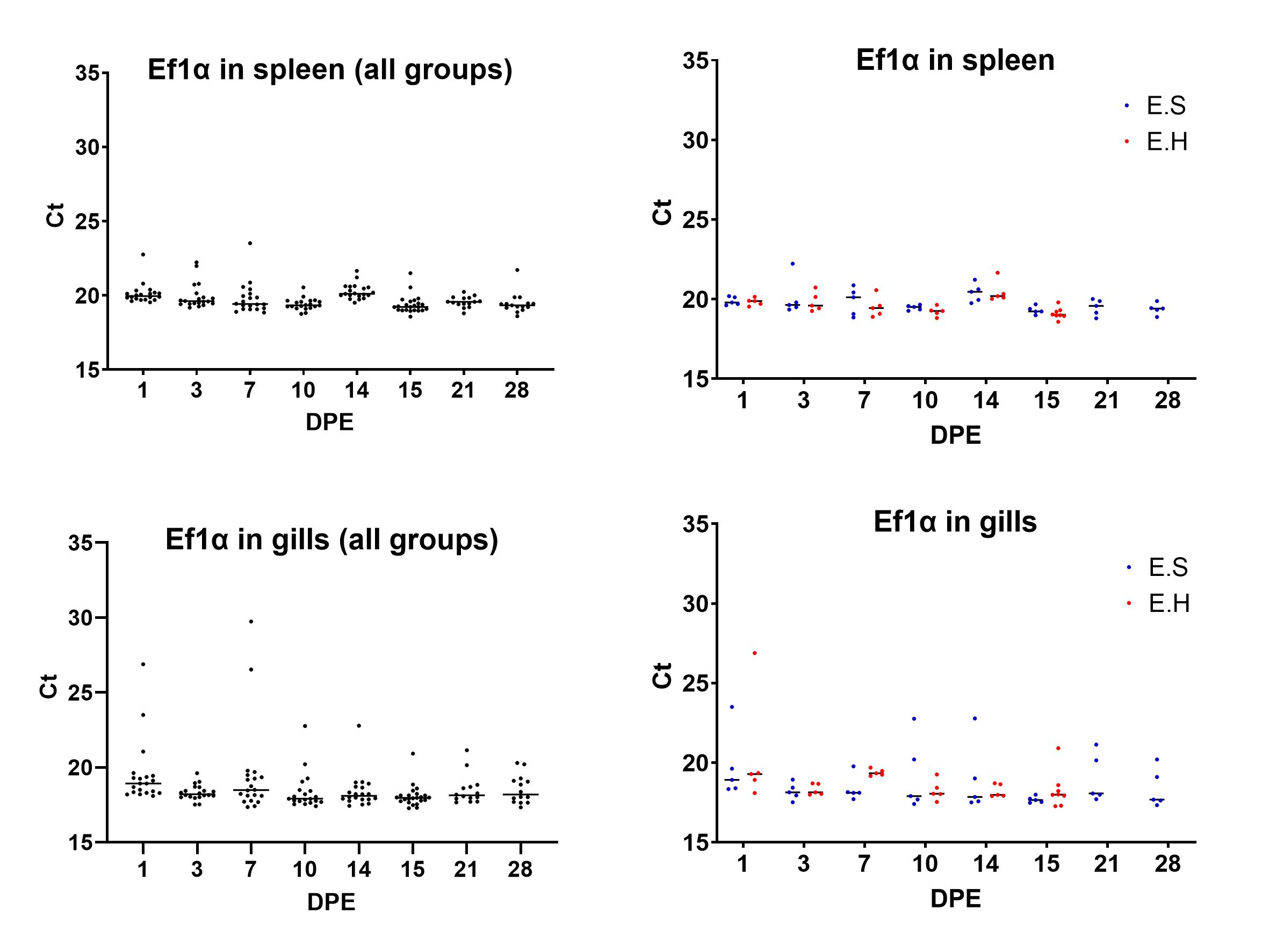

Supplement: Supplementary file 2 [file Image_2.jpg]
