# Supplementary material for: Mucosal and Systemic Immune Responses to Salmon Gill Poxvirus Infection in Atlantic Salmon Are Modulated Upon Hydrocortisone Injection
Source: Front Immunol. 2021 Jun 9;12:689302. doi: 10.3389/fimmu.2021.689302 (PMC8221106; doi:10.3389/fimmu.2021.689302)

In situ hybridization with probes targeting B22R1 and D13L

RNAscope 2.5 HD Detection kit – RED

L53, 1 DPE, RNAscope probe: B22R1 (ct - 33,4):

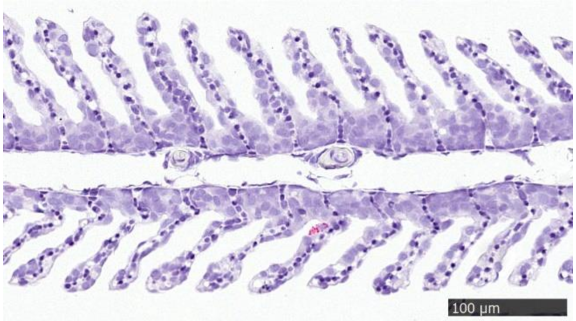

L53, 1 DPE, RNAscope probe: D13L (ct - 37,3):

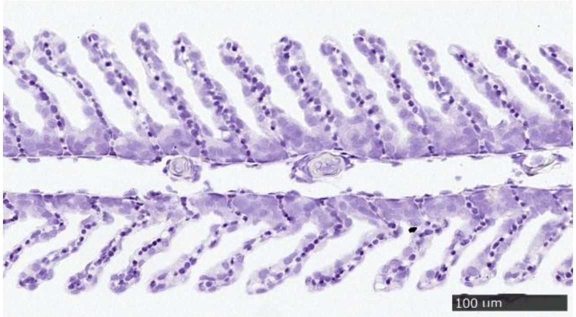

L54, 1 DPE, RNAscope probe: B22R1 (ct - 31,1):

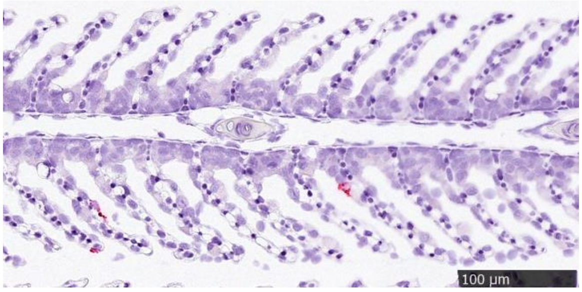

L54, 1 DPE, RNAscope probe: D13L (ct - 35,9):

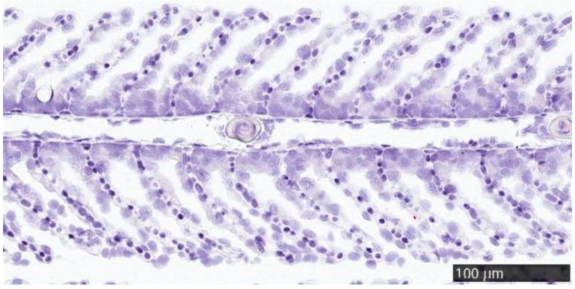

L55, 1 DPE, RNAscope probe: B22R1 (ct - 30,4):

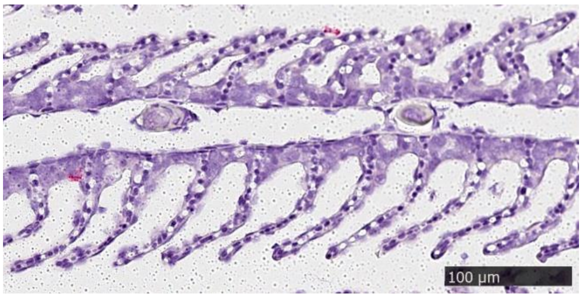

L53, 1 DPE, RNAscope probe: D13L (ct - 34,6):

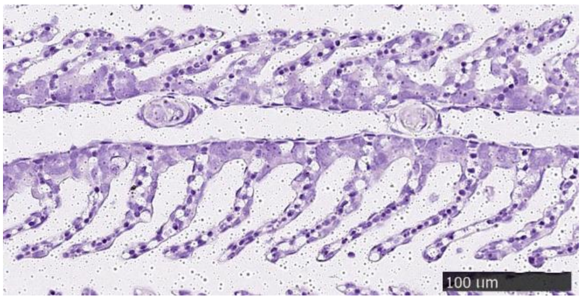

L77, 3 DPE, RNAscope probe: B22R1 (ct - 28,6):

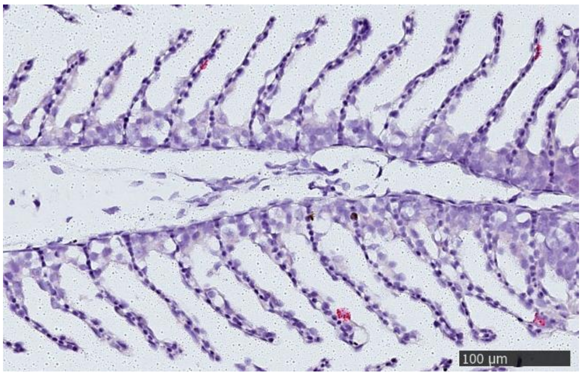

L77, 3 DPE, RNAscope probe: D13L (ct - 24,7):

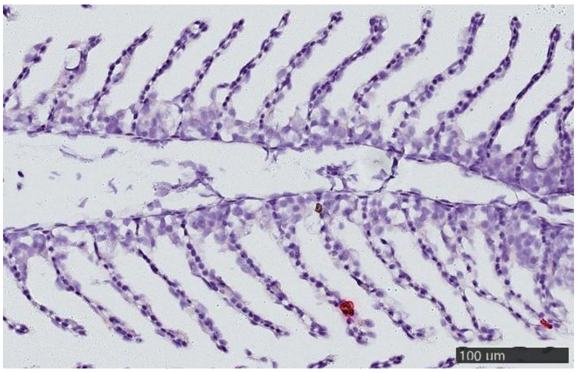

Supplement: Supplementary file 3 [file Image_3.pdf]
