# Supplementary material for: Mucosal and Systemic Immune Responses to Salmon Gill Poxvirus Infection in Atlantic Salmon Are Modulated Upon Hydrocortisone Injection
Source: Front Immunol. 2021 Jun 9;12:689302. doi: 10.3389/fimmu.2021.689302 (PMC8221106; doi:10.3389/fimmu.2021.689302)

In situ hybridization with probes targeting D13L and GzmA at 7 DPE

RNAscope 2.5 HD Duplex Detection kit (Chromogenic)

E.S, L93, GzmA (ct - 26,9) and D13L (ct - 25,1):

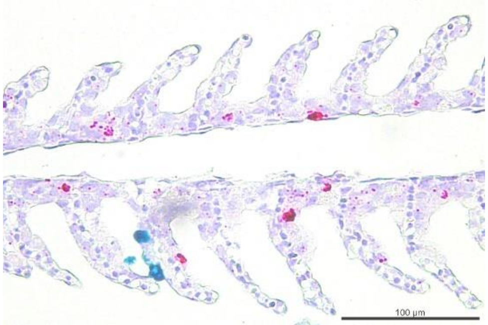

E.S, L94, GzmA (ct - 28,7) and D13L (ct - 28,5):

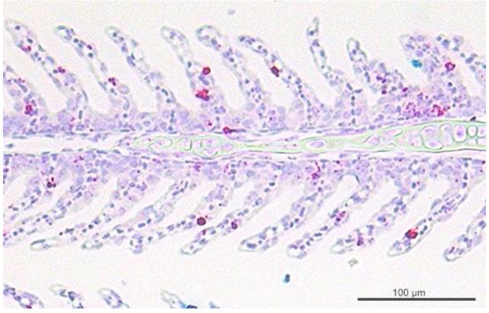

E.S, L95, GzmA (ct - 26,5) and D13L (ct - 26,3):

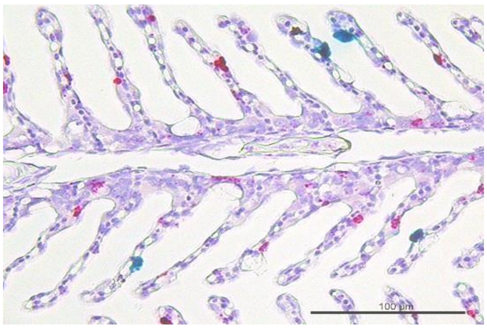

E.H, L98, GzmA (ct - 32,9) and D13L (ct - 24,0):

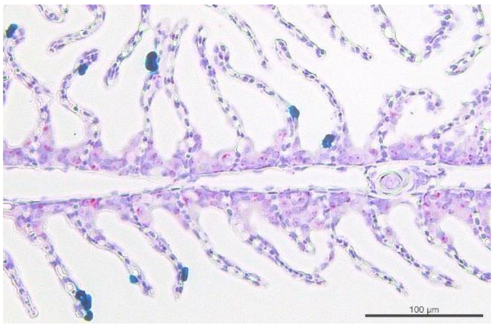

E.H, L100, GzmA (ct - 31,7) and D13L (ct - 27,7):

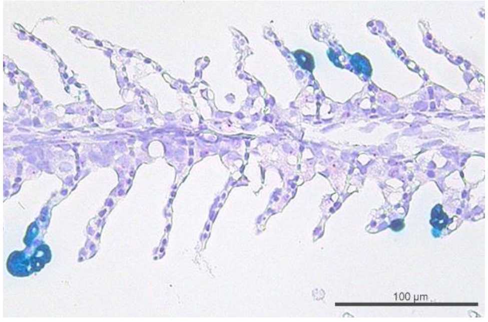

E.H, L101, GzmA (ct - 31,4) and D13L (ct - 24,0):

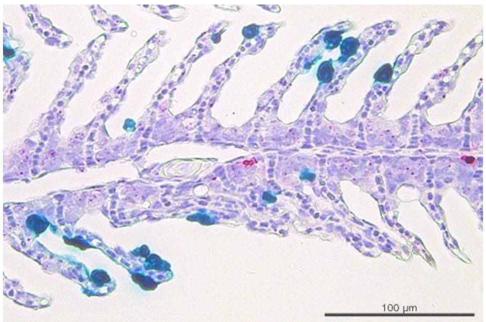

C.S, L85, GzmA (ct - 28,8) and D13L (ct - N/A):

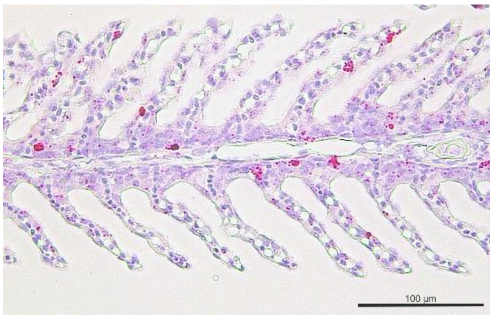

C.H, L89, GzmA (ct - 32,1) and D13L (ct - N/A):

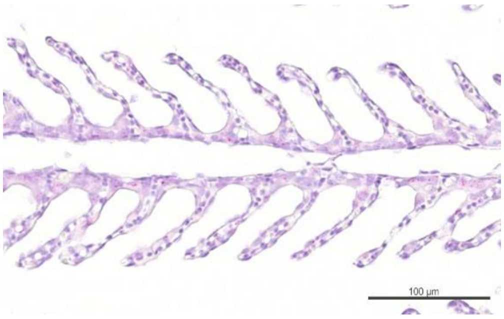

Supplement: Supplementary file 4 [file Image_4.pdf]
