# Supplementary material for: Mucosal and Systemic Immune Responses to Salmon Gill Poxvirus Infection in Atlantic Salmon Are Modulated Upon Hydrocortisone Injection
Source: Front Immunol. 2021 Jun 9;12:689302. doi: 10.3389/fimmu.2021.689302 (PMC8221106; doi:10.3389/fimmu.2021.689302)

In situ hybridization with probes targeting D13L and GzmA at 14 DPE

RNAscope 2.5 HD Duplex Detection kit (Chromogenic)

E.S, L134, GzmA (ct - 30,7) and D13L (ct - N/A):

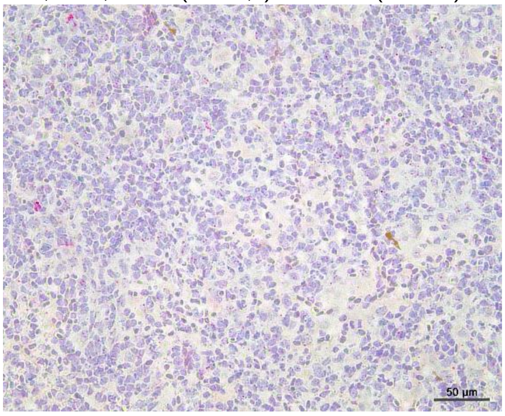

E.S, L137, GzmA (ct - 31,7) and D13L (ct - N/A):

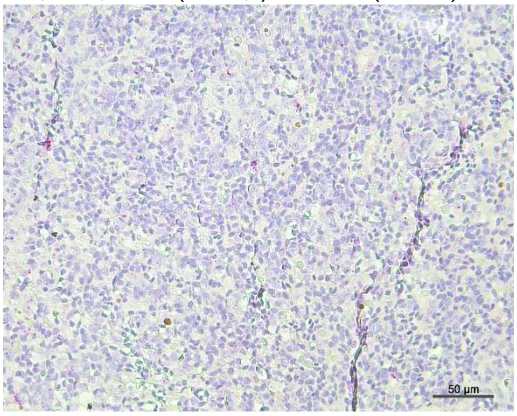

E.S, L138, GzmA (ct - 31,0) and D13L (ct - N/A):

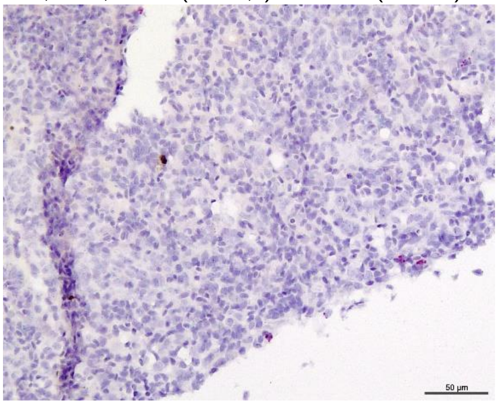

E.H, L139, GzmA (ct - 27,8) and D13L (ct - 36,4):

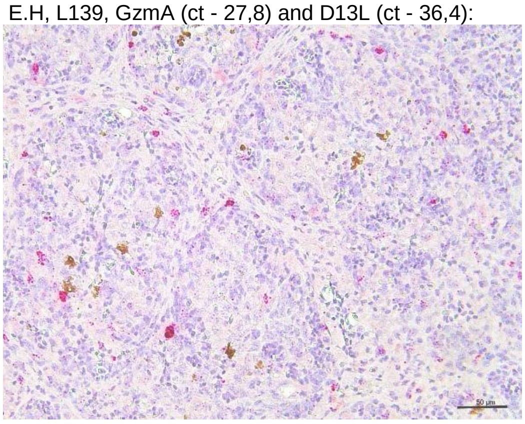

E.H, L140, GzmA (ct - 28,0) and D13L (ct - N/A):

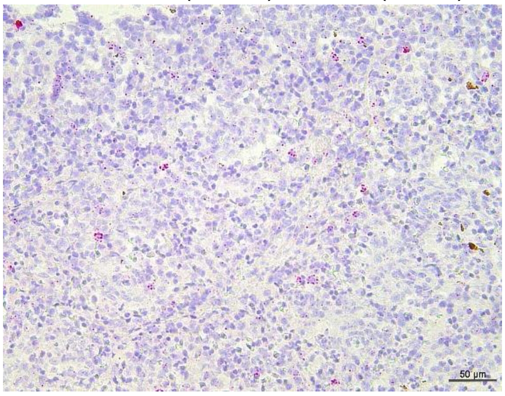

E.H, L141, GzmA (ct - 26,1) and D13L (ct - N/A):

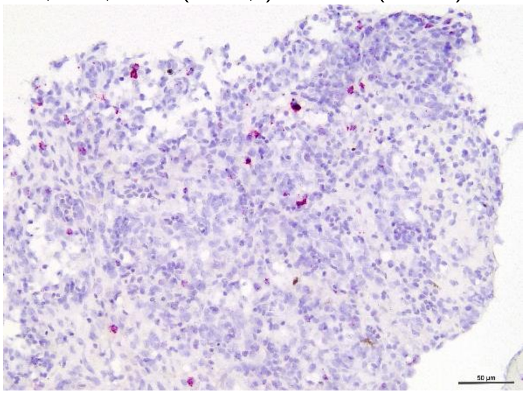

C.S, L128, GzmA (ct - 31,4) and D13L (ct - N/A):

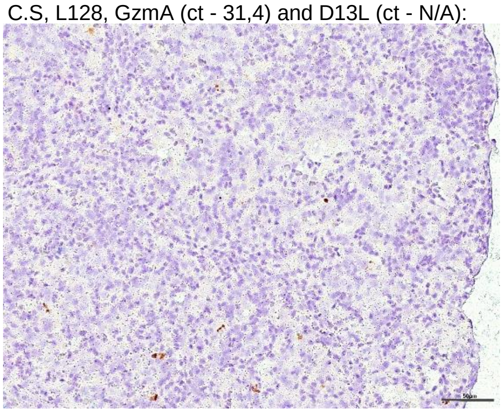

C.H, L132, GzmA (ct - 29,1) and D13L (ct - N/A):

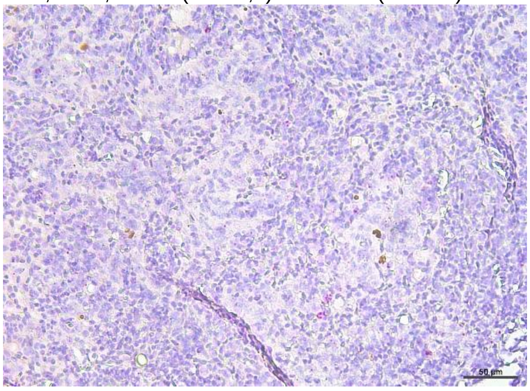

Supplement: Supplementary file 5 [file Image_5.pdf]
